# Minimally Invasive Surgery in the Management of Advanced Epithelial Ovarian Cancer: A Comprehensive Analysis of Current Evidence and Clinical Applications

**DOI:** 10.3390/medicina61122201

**Published:** 2025-12-12

**Authors:** Filippo Alberto Ferrari, Matteo Pavone, Ilaria Cuccu, Federico Ferrari, Giorgio Bogani, Marcello Ceccaroni

**Affiliations:** 1 Department of Obstetrics and Gynecology, Gynecologic Oncology and Minimally Invasive Pelvic Surgery, International School of Surgical Anatomy (ISSA), IRCCS “Sacro Cuore-Don Calabria” Hospital, Negrar di Valpolicella, 37024 Verona, Italy; 2 UOC Ginecologia Oncologica, Dipartimento di Scienze per la Salute della Donna e del Bambino e di Sanità Pubblica, Fondazione Policlinico Universitario A. Gemelli, IRCCS, 00168 Rome, Italy; matteopavone.21@gmail.com; 3 IHU Strasbourg, Institute of Image-Guided Surgery, 67091 Strasbourg, France; 4 Research Institute Against Digestive Cancer (IRCAD), 67091 Strasbourg, France; 5 Gynecological Oncology Unit, Fondazione IRCCS Istituto Nazionale dei Tumori, 20133 Milano, Italy; ilaria.cuccu@uniroma1.it (I.C.); bogani.giorgio@gmail.com (G.B.); 6 Department of Clinical and Experimental Sciences, University of Brescia, 25121 Brescia, Italy; federico.ferrari@unibs.it

**Keywords:** ovarian cancer, laparoscopic surgery, interval debulking surgery

## Abstract

*Background and Objectives*: Advanced epithelial ovarian cancer (AEOC) often requires extensive cytoreductive surgery. Minimally invasive surgery (MIS), especially diagnostic laparoscopy, is increasingly used to assess resectability and guide treatment. This review aimed to evaluate the evidence on MIS in AEOC, focusing on its diagnostic and therapeutic roles in primary and interval debulking surgery (PDS and IDS), and its impact on perioperative and oncologic outcomes. *Materials and Methods*: A structured literature review was performed using PubMed, MEDLINE, Embase, Scopus, and the Cochrane Library, including studies published between January 2000 and June 2025. Eligible studies involved laparoscopic or minimally invasive cytoreduction in PDS or IDS, reporting surgical feasibility, perioperative results, and oncologic outcomes. Data were synthesized qualitatively due to heterogeneity across studies. *Results*: Observational studies indicate that diagnostic laparoscopy predicts resectability, reduces futile laparotomies, and improves patient selection for primary surgery. In selected patients, non-randomized cohorts of laparoscopic PDS report R0 resection rates up to 95%, with low morbidity and short hospital stays. In IDS after neoadjuvant chemotherapy, MIS has been associated with reduced blood loss, fewer complications, and faster postoperative recovery, while showing progression-free and overall survival comparable to laparotomy in retrospective series. Conversion to open surgery was generally reported in fewer than 10% of cases when stringent selection criteria were applied. *Conclusions*: Diagnostic laparoscopy is a valuable tool for accurate preoperative evaluation and surgical planning in EOC. MIS, particularly for IDS, appears to offer reduced morbidity and equivalent survival outcomes when performed in experienced centers, whereas its application in PDS remains investigational and should be reserved for highly selected cases. These conclusions are limited by the predominance of retrospective evidence and the heterogeneity in patient selection and surgical expertise.

## 1. Introduction

Epithelial ovarian cancer (EOC) remains one of the most lethal gynecologic malignancies globally, representing the eighth most common cancer among women and the leading cause of death from gynecologic tumors [1]. Its high mortality rate is primarily attributable to its insidious onset and lack of specific early symptoms, which result in the majority of patients being diagnosed at an advanced stage (FIGO stage III or IV) [1,2]. At this point, disease dissemination within the peritoneal cavity is typically extensive, and therapeutic management becomes more complex. Despite notable advancements in systemic therapies, including the introduction of targeted agents such as PARP inhibitors and antiangiogenic drugs, the five-year overall survival (OS) for patients with advanced-stage EOC remains below 45% [3,4].

The cornerstone of treatment for advanced EOC continues to be a multimodal strategy consisting of cytoreductive surgery followed by platinum-based chemotherapy. Among prognostic factors, the amount of residual disease after surgery is the most critical determinant of both progression-free survival (PFS) and OS [5]. Numerous studies have established that patients who achieve complete cytoreduction, defined as no visible residual disease (R0), have significantly better outcomes compared to those with macroscopic residual tumor. Traditionally, primary debulking surgery (PDS) was considered the gold standard and was performed upfront in all patients deemed operable. However, in the last two decades, a paradigm shift has occurred with the increasing use of neoadjuvant chemotherapy (NACT) followed by interval debulking surgery (IDS) [6]. This approach was initially reserved for patients with high tumor load, extensive comorbidities, or poor performance status who were unlikely to benefit from aggressive primary surgery. Subsequent randomized clinical trials, such as EORTC-55971 and CHORUS, as well as large meta-analyses, have demonstrated non-inferior oncologic outcomes for IDS compared to PDS [7,8]. As a result, NACT followed by IDS is now widely accepted as an alternative standard of care in appropriately selected cases, and international guidelines support its use in patients unlikely to achieve optimal debulking through PDS.

In parallel with these developments, there has been a growing interest in the use of minimally invasive surgery (MIS) in gynecologic oncology [9]. MIS has become the preferred surgical approach for early-stage endometrial and ovarian cancer. Its benefits, including reduced intraoperative blood loss, fewer postoperative complications, shorter hospital stays, and quicker return to normal activities, have been extensively documented and are now widely accepted in clinical practice [10,11]. These advantages have prompted consideration of MIS in the management of advanced EOC. The cytoreductive procedures required for EOC are complex, frequently involving multiquadrant surgery, adhesiolysis, and, in many cases, upper abdominal resections [12]. These factors raise concerns about the technical feasibility of MIS and its oncologic safety, including the ability to achieve R0 resections and avoid atypical recurrence patterns. Moreover, MIS may be associated with longer operative times and requires specific surgical expertise and institutional infrastructure [13].

Beyond its therapeutic implications, MIS has also emerged as a critical diagnostic tool in surgical planning. In accordance with ESGO–ESMO–ESP and NCCN guidelines, diagnostic laparoscopy may be used when incomplete cytoreduction is suspected on imaging, helping to avoid unnecessary laparotomies in those unlikely to benefit from primary surgery and minimally invasive approaches are acceptable for apparent stage I disease when performed by experienced gynecologic oncologists [14,15]. Scoring systems such as the Fagotti score have demonstrated strong predictive value for complete cytoreduction and are recommended by international guidelines [14]. Recent evidence also underscores the feasibility of robotic surgery in selected cases of recurrent ovarian cancer undergoing secondary cytoreduction. Beyond showing perioperative and oncologic outcomes comparable to laparoscopy, the robotic platform offers distinct technical advantages, including 360° instrument mobility, tremor filtration, stable 3D visualization, and improved surgeon ergonomics, which may enhance precision in appropriately selected patients [16,17].

This review aims to critically appraise the current literature on the role of MIS in the surgical management of advanced EOC, evaluating the role of laparoscopy in the setting of primary debulking surgery, both as a preoperative diagnostic tool and as a therapeutic option, in carefully selected patients in PDS or IDS. By synthesizing data from observational studies, institutional experiences, and early prospective trials, this review aims to provide a clinically relevant framework that supports evidence-based decision-making and identifies areas where future research is most needed.

## 2. Materials and Methods

A structured and comprehensive literature review was carried out to evaluate the role of MIS in both interval and primary cytoreductive procedures for advanced EOC. Relevant studies were identified in multiple databases, including PubMed, MEDLINE, Embase, Scopus, and Cochrane Library. The search covered literature published from January 2000 to May 2025. An additional manual review of the reference lists from selected studies was conducted to identify any potentially eligible articles not retrieved during the initial search. The search strategy incorporated a combination of medical subject headings (MeSH) and keywords, including: “minimally invasive surgery,” “laparoscopy,” “diagnostic laparoscopy,” “interval debulking surgery,” “primary debulking surgery,” “advanced epithelial ovarian cancer,” “neoadjuvant chemotherapy”, “cytoreductive surgery,” and “ovarian cancer recurrence.” Studies were considered eligible if they included patients undergoing laparoscopic approach for either PDS and IDS and reported on at least one of the following: surgical feasibility, perioperative outcomes (e.g., operative time, blood loss, complications, length of hospital stay), conversion rates, or oncologic endpoints (e.g., rate of complete cytoreduction, progression-free survival, overall survival). We included retrospective and prospective cohort studies as well as randomized controlled trials evaluating MIS or laparoscopy in primary or interval debulking surgery. Only full-text, peer-reviewed articles published in English were considered eligible. Case reports, expert opinions, conference abstracts without full-text availability, and editorials were excluded. Publications were screened independently by multiple reviewers (FAF, MP, IC). Each reviewer was assigned a subset of articles for title and abstract screening, followed by full-text analysis. In instances where eligibility was unclear or disagreements arose regarding inclusion, final decisions were reached by consensus after consultation with two senior authors (MC, FG). Given the anticipated heterogeneity across studies in terms of patient selection, surgical techniques, and reported endpoints, the data synthesis was conducted in a qualitative manner. The features of the relevant included studies are summarized in Table 1.

## 3. Results

### 3.1. Role of Laparoscopic Surgery in Primary Debulking and in Its Planning

PDS remains the standard surgical approach for advanced EOC, with complete cytoreduction representing the most significant prognostic factor for improving patient survival [6]. However, complete macroscopic resection is not achieved in a considerable proportion of patients due to widespread intra-abdominal disease or patient-related factors that limit the feasibility of ultra-radical procedures [12]. To avoid unnecessary laparotomies in cases where optimal debulking is unlikely, considerable efforts have been directed toward refining preoperative assessment and surgical planning strategies [34].

Diagnostic laparoscopy has emerged as an essential tool in this context. Since the early 2000s, staging laparoscopy has been employed to evaluate the resectability of disease before PDS [14,18]. Pioneered by Vergote et al. and later systematized by Fagotti et al., the use of laparoscopic triage has reduced the incidence of futile laparotomies [14]. The Fagotti predictive index value score has demonstrated high specificity and reproducibility, particularly when a threshold of ≥8 is used to predict suboptimal cytoreduction [14]. These findings have been confirmed in multicenter prospective trials, such as the Olympia-MITO 13 study, which validated the external applicability of the score across different surgical settings [19]. More recently, other authors have confirmed that diagnostic laparoscopy reduces the rate of suboptimal cytoreduction in the PDS setting and decreases the use of NACT among non-HGSC patients. It also minimized postoperative complications while having no impact on overall survival [30]. Vizzielli et al. demonstrated that diagnostic laparoscopy serves not only to assess tumor resectability but also to predict the risk of major postoperative complications following primary debulking surgery in advanced ovarian cancer. By developing and validating a laparoscopic scoring system, the study highlights the pivotal role of laparoscopy in preoperative patient selection and individualized surgical planning [22]. Although this tool demonstrated good predictive accuracy, it is not without limitations. Its performance is reduced in patients with extensive adhesions, bulky masses, or ascites, which can obscure visualization and hinder full peritoneal assessment [19,35]. Furthermore, staging laparoscopy has limited ability to assess disease in retroperitoneal or retrohepatic regions, which can result in an underestimation of overall tumor spread. Reported complication rates range from 1% to 5%, and although uncommon, port-site metastases have been described in up to 3% of cases, particularly in patients with significant ascites [34]. As a result, the adoption of staging laparoscopy differs among institutions and is strongly influenced by surgeon expertise and local practice standards. In high-volume centers, however, it is increasingly incorporated as a routine component of preoperative evaluation to refine treatment planning and avoid unnecessary exposure to neoadjuvant chemotherapy [13,36].

Beyond its role in planning, MIS has also been proposed in selected cases as the definitive approach for performing PDS. In carefully selected patients with favorable imaging findings and adequate performance status, laparoscopic cytoreduction has been shown to achieve comparable oncologic outcomes with reduced morbidity. Ceccaroni et al. explored this approach in a prospective cohort of patients with presumed FIGO stage III–IV disease, who were preoperatively evaluated with PET/CT and pelvic ultrasound. Only patients without radiologic evidence of bulky upper abdominal disease were considered for laparoscopic PDS. The study demonstrated high complete resection rates and low intraoperative complication rates, highlighting the feasibility of this strategy in a strictly selected population [37].

In a subsequent retrospective study published in 2023, Ceccaroni et al. refined the definition of selected patients by specifying inclusion criteria that excluded individuals with extensive omental, diaphragmatic, splenic, or multivisceral involvement, as well as those requiring more than two bowel resections [13]. Among the selected patients who underwent laparoscopic PDS, the R0 rate was 95%, with minimal perioperative complications and a median hospital stay of five days. Importantly, no compromise in oncologic outcomes was observed, further reinforcing that laparoscopic PDS can be safely performed in experienced centers when rigorous selection protocols are applied [13]. Complementary data were provided by Tozzi et al. through the ULTRA-LAP study, which investigated laparoscopic visceral-peritoneal debulking as a structured surgical method in both PDS and IDS settings [13]. According to Tozzi, patients who may be considered ‘selected’ and therefore potential candidates for a laparoscopic approach in primary debulking surgery are those with confirmed or suspected FIGO stage IIIC–IV disease, an ECOG performance status ≤ 2, and evidence of response or stable disease to neoadjuvant chemotherapy. Importantly, candidates should be excluded when preoperative assessment reveals lung metastases, involvement of three or more liver segments, or disease progression, and likewise excluded intraoperatively when diffuse small-bowel serosal deposits or porta hepatis encasement are identified. Although the majority of patients included in the ULTRA-LAP series underwent interval debulking, the standardized laparoscopic techniques described, such as diaphragmatic peritonectomy, multi-quadrant peritoneal stripping, and mesenteric resection, underscore the expanding scope of laparoscopy in advanced ovarian cancer. The reproducibility and safety of these complex procedures, when performed in specialized centers, offer potential applicability to select PDS cases as well [13].

### 3.2. Laparoscopy in Interval Debulking Surgery

MIS for IDS has emerged as a promising approach in the treatment of advanced EOC, particularly in patients selected based on favorable biological and radiological response to NACT [13]. Evidence from multiple observational and prospective studies supports its feasibility, safety, and oncologic non-inferiority compared to traditional open surgery, provided that strict selection criteria are applied and procedures are performed in specialized, high-volume centers. The INTERNATIONAL MISSION, MISSION trial, and other series retrospectively have confirmed the feasibility of using minimally invasive surgery in the interval debulking setting [11,21,24,31].

In the literature, patient selection for MIS-IDS has consistently been guided by clinic-radiological criteria. Complete or partial radiologic response to NACT, as assessed by RECIST or GCIG criteria via abdominal/pelvic CT, along with a significant reduction in serum CA-125 levels, was universally adopted as baseline indicators of eligibility. Several prospective trials, including the CILOVE [28], MIRRORS [33], and ULTRA-LAP [12] studies, further incorporated diagnostic laparoscopy with application of the Fagotti score to objectively evaluate peritoneal resectability, enhancing preoperative risk stratification and surgical planning.

Operative outcomes have consistently favored MIS in well-selected IDS cohorts. In a prospective multicentre study, Gueli Alletti et al. reported significantly lower estimated blood loss (median 100 mL) and shorter hospital stay (median 4 days) in the MIS group compared to laparotomy, with a higher rate of early chemotherapy resumption (20 vs. 35 days, *p* = 0.003) [21]. Similarly, in a retrospective single-center study, Ceccaroni et al. described a median operative time of 275 min and blood loss of 150 mL in laparoscopic IDS, with a complete cytoreduction (R0) rate of 95%, confirming the feasibility of extensive procedures via MIS in selected patients [13]. In the CILOVE trial, a multicenter prospective study, R0 resection was achieved in 97% of patients undergoing laparoscopic IDS, with no grade ≥ 3 complications, and a median length of stay of five days [28], and a similarly high complete resection rate was reported in the LANCE pilot study [32].

Conversion to open surgery is a critical indicator of MIS safety, and its likelihood is closely tied to the rigor of preoperative assessment. Studies applying structured selection protocols, including staging laparoscopy or validated prediction models, consistently report conversion rates below 10%, reflecting the effectiveness of identifying suitable candidates [10,20,21,24,26,27]. In contrast, series adopting broader inclusion criteria show substantially higher rates, reaching up to 23%, largely driven by intraoperative findings that were not apparent preoperatively, such as diffuse peritoneal carcinomatosis, multifocal bowel involvement, or mesenteric retraction [25]. These patterns highlight that incomplete preoperative evaluation increases the risk of encountering disease patterns incompatible with MIS, underscoring the need for meticulous imaging review, objective scoring systems, and, when appropriate, diagnostic laparoscopy to minimize unexpected conversions [25].

Regarding oncologic outcomes, multiple studies have demonstrated that MIS can achieve comparable survival rates to open surgery in selected patients [21,24,29,32]. For example, Gueli Alletti et al. reported a significantly longer PFS in the MIS group (18 vs. 12 months, *p* = 0.027), and Ackroyd et al., in a retrospective series, described a median PFS and OS of 21.2 and 39.7 months, respectively [23]. The retrospective MISSION trial reported a median PFS of 23 months and a five-year OS rate of 52.6%, with recurrence patterns largely peritoneal and not suggestive of atypical dissemination due to MIS [21]. Data from the National Cancer Database supported these findings at scale, revealing improved OS in patients undergoing MIS (35.9 vs. 34.5 months, *p* = 0.01), with a 14% reduction in the hazard of all-cause mortality (HR = 0.86, 95% CI: 0.79–0.94) [31]. Notably, the study controlled for selection bias through multivariate analysis and matched cohorts, lending weight to the observed survival benefit. Importantly, the ability to achieve R0 resection remains central to the oncologic safety of MIS. Brown et al. d Jørgensen et al. retrospectively found no significant difference in R0 rates between MIS and laparotomy (91% vs. 86% and 90% vs. 88%, respectively) [24,31], while Davidson et al. observed a lower R0 rate in the MIS group (74% vs. 92%, *p* = 0.03), likely reflecting broader eligibility and less stringent preoperative evaluation [25]. These findings underscore the need for robust selection protocols to mitigate the risk of suboptimal cytoreduction in MIS.

## 4. Discussion

Our study offers a comprehensive narrative synthesis of current evidence regarding the use of MIS in the treatment of advanced EOC. Although laparotomy remains the standard of care in the majority of cytoreductive procedures for advanced EOC, the adoption of MIS has expanded in recent years, driven by its potential perioperative advantages and the growing body of literature supporting its safety in selected clinical contexts.

In the context of IDS, we found that MIS is a viable approach in highly selected patients with a favorable response to NACT and absence of extensive disease in critical anatomical sites [24,25,32]. The rationale for this approach is rooted in the principle of tumor downstaging through NACT, which may enable complete cytoreduction via less invasive means. As demonstrated by many authors, proper patient selection using clinical, radiological, and laparoscopic criteria is fundamental to achieving optimal outcomes with MIS [12,13,21,28]. These criteria include partial or complete radiological response, CA-125 normalization, and, in many cases, diagnostic laparoscopy guided by scoring systems such as the Fagotti score to assess disease distribution and resectability [14].

Perioperative outcomes are consistently favorable in MIS-IDS cohorts. Blood loss is significantly reduced compared to open surgery, often falling below 200 mL, while hospital stay is shortened to a median of 3–5 days in most studies. Additionally, MIS appears to facilitate faster recovery and earlier resumption of adjuvant chemotherapy—a factor associated with improved prognosis, especially in high-grade serous tumors [10,11,20]. Brown et al. and Gueli Alletti et al. both reported significantly shorter times to chemotherapy in the MIS group compared to laparotomy [21,24], a finding also observed by Ceccaroni et al. [37]. Importantly, these perioperative benefits do not seem to come at the expense of oncologic efficacy. Most of the included studies report comparable R0 resection rates between MIS and open surgery, ranging from 85% to 97%, depending on patient selection and surgical expertise.

Oncologic outcomes, while still limited by the retrospective design of most studies, are encouraging. Several analyses have shown no significant differences in PFS or OS between MIS and open approaches. A National Cancer Database study reported a modest but statistically significant OS advantage in patients treated with MIS (35.9 vs. 34.5 months, *p* = 0.01), after adjusting for confounders [31]. Similarly, the MISSION trial showed a 6-month increase in PFS in the MIS group and a 5-year OS rate of over 50%, consistent with outcomes typically observed in open cohorts [21]. These findings support the oncologic safety of MIS when used in well-selected patients and reinforce the central role of complete cytoreduction as the primary driver of survival, regardless of surgical approach.

Despite these promising results, several limitations must be acknowledged. First and foremost, the available evidence is largely derived from retrospective or non-randomized prospective studies, with inherent risks of selection bias. Patients undergoing MIS were typically younger, had better performance status, lower tumor burden, and greater response to chemotherapy, factors independently associated with improved outcomes [6,7,32]. Moreover, surgical expertise and institutional volume likely played a critical role in influencing results. The studies by Ceccaroni et al. and Tozzi et al. were conducted in specialized centers with high levels of laparoscopic proficiency and structured protocols, generalizing to lower-volume institutions [12,13,37]. Importantly, the learning curve for complex MIS in ovarian cancer is substantial, requiring dedicated training, high case volumes, and progressive exposure to advanced cytoreductive techniques to ensure safety and reproducibility. Additionally, the lack of standardized reporting on surgical complexity, intraoperative complications, and recurrence patterns limits the interpretability of the data. While no increase in port-site metastases or atypical recurrence has been documented in these studies, the long-term safety of MIS in the management of disseminated peritoneal disease requires further validation through robust RCTs [38]. The ongoing LANCE and MIRRORS-RCT studies are expected to provide higher-level evidence regarding the safety and efficacy of MIS in IDS, potentially informing future guidelines and expanding indications in broader clinical practice [33,39].

In parallel, the role of MIS in the context of primary debulking surgery remains more restricted. While traditionally reserved for diagnostic purposes (particularly in surgical triage between PDS and NACT), laparoscopy has also been explored as a definitive therapeutic approach in selected PDS cases [9,14]. Laparoscopic PDS may be feasible in patients with limited tumor burden and favorable anatomical distribution, as determined by cross-sectional imaging and multidisciplinary evaluation. When performed in experienced centers, MIS-PDS has yielded R0 rates of up to 95%, with low complication rates and acceptable operative times [12,13]. The mainstay of PDS continues to be laparotomy, particularly in the presence of diaphragmatic, mesenteric, or extensive upper abdominal disease, where multivisceral resections are often required [35]. A number of limitations should be acknowledged when interpreting these findings. The current evidence relies largely on retrospective studies conducted in single centers, which makes it difficult to draw reliable conclusions and reduces generalizability. There is also substantial heterogeneity in patient selection, surgical techniques, and institutional experience, and high-volume centers with dedicated MIS programs are more likely to report favorable outcomes. Selection bias is an additional concern, as patients offered MIS often have better performance status, more favorable disease distribution, or lower tumor burden, which may inadvertently magnify the apparent advantages of minimally invasive approaches. Publication bias may further influence the literature by favoring studies with positive or reassuring results. Finally, the lack of large, rigorously designed randomized trials, particularly for MIS in primary debulking surgery, remains a major gap and continues to constrain the strength of current recommendations. These limitations highlight the need for cautious interpretation of the available data and reinforce the importance of future prospective, multicenter research. In conclusion, MIS for IDS represents a safe and effective option for a well-defined subset of patients with advanced ovarian cancer who demonstrate a favorable response to NACT and limited residual disease. Its use is associated with improved perioperative outcomes and does not appear to compromise oncologic efficacy when performed in expert hands. Nonetheless, rigorous patient selection, standardized protocols, and high institutional expertise are critical for its safe implementation. The role of MIS in PDS remains more investigational and should be reserved for highly selected cases within specialized centers. Future research must focus on expanding prospective evidence, defining standardized selection tools, and clarifying the long-term oncologic safety of MIS in this high-stakes surgical domain.

## 5. Conclusions

Minimally invasive surgery plays an increasingly relevant role in the management of advanced epithelial ovarian cancer. Diagnostic laparoscopy is a key tool for assessing tumor resectability, optimizing patient selection, and reducing unnecessary laparotomies. In selected cases, MIS (particularly in interval debulking surgery) provides comparable oncologic outcomes to open surgery with significant perioperative advantages. Its application in primary debulking surgery remains investigational, supported by limited data in highly selected patients, and should be reserved for specialized centers with extensive expertise in advanced gynecologic MIS. To better define the appropriate indications, safety profile, and long-term oncologic impact of MIS in both IDS and PDS, high-quality prospective studies and well-designed randomized trials are urgently needed.

## Figures and Tables

**Table 1:** Summary of relevant included studies.

Authors	Year	Country	Study Objective	Study Type	N	Intervention	Key Findings	Significant Oncologic Outcomes
Fagotti et al. [14]	2005	Italy	MIS vs. laparotomy in assessing the chance of optimal cytoreduction	Prospective	61	Laparoscopic assessment IDS	In the final model, a predictive index score > or =8 identified patients undergoing suboptimal surgery with a specificity of 100%	Not evaluated
Brun et al. [18]	2008	France	MIS vs. laparotomy in assessing the chance of optimal cytoreduction	Retrospective	55	Laparoscopic assessment PDS	The proposed laparoscopic 7 items score demonstrated a sensitivity, specificity, PPV, NPV, and accuracy of 46%, 89%, 89%, 44%, and 60% to predict resectability	Not evaluated
Fagotti et al. (Olympia-MITO13) [19]	2013	Italy	Role of MIS in assessing the chance of optimal cytoreduction	Prospective multicenter	168	Laparoscopic assessment PDS	The worst laparoscopic feature was mesenteric retraction; Cohen’s kappa and the *p* value for overall predictive index value were 0.685 and 0.01	Not evaluated
Gueli Alletti et al. [20]	2016	Italy	Compare MIS vs. laparotomy	Retrospective case–control	95	Laparoscopic IDS	Less blood loss, shorter stay with MIS	Better PFS with Bevacizumab; OS comparable in MIS group
Gueli Alletti et al. (MISSION) [21]	2016	Italy	Feasibility of laparoscopic IDS	Prospective feasibility	52	Laparoscopic IDS	Minimal complications, high feasibility	Not evaluated
Vizzielli et al. [22]	2016	Italy	Laparoscopic score to predict major postoperative complications after PDS	Retrospective multicenter	555	Laparoscopic assessment PDS	High laparoscopic tumor load was a significant predictor	Not evaluated
Ackroyd et al. [23]	2017	USA	Feasibility of robotic IDS	Retrospective single-center	29	Robotic IDS	R0: 66%; shorter hospital stay	PFS: 21.2 mo; OS: 39.7 mo
Brown et al. [24]	2019	USA	Compare MIS and laparotomy	Retrospective comparative	157	Laparoscopic IDS	Higher R0 in MIS; shorter stay	R0 higher in MIS; OS/PFS not reported
Davidson et al. [25]	2019	Canada	Complexity score and survival outcomes	Retrospective multicenter	282	Laparoscopic IDS + complexity score	R0: 76%; higher complexity = worse DSS	R0: 76%; OS/PFS not reported
Fagotti et al. (INTERNATIONAL MISSION) [11]	2019	Italy	Evaluate MIS safety after NACT	Retrospective multicenter	127	Laparoscopic IDS	R0: 96.1%; major complications < 5%	PFS: 23 mo; OS at 5 yrs: 52%
Zhang et al. [26]	2021	China	Compare robotic and open surgery + impact of NACT cycles	Retrospective	93	Robotic IDS	R0: 63% vs. 52%; fewer complications in robotic group	PFS: 16.7 vs. 15.4 mo; OS: 35.6 vs. 38.2 mo
Morton et al. [27]	2021	USA	Compare MIS vs. open surgery + HIPEC	Retrospective	50	MIS IDS + HIPEC	R0: 70% vs. 77.5%; shorter stay with MIS	PFS: 15 (MIS) vs. 17.2 (open); OS not reported
Pomel et al. (CILOVE) [28]	2021	France	Feasibility of laparoscopic IDS in NACT responders	Prospective phase II	48	Laparoscopic IDS	R0: 97%; low conversion and complication rates	PFS: 87.5% at 12 months
Lecointre et al. [29]	2022	France	Compare MIS and laparotomy	Retrospective propensity-score-matched	37	Laparoscopic IDS	Less early postoperative complications (6 versus 17, *p* = 0.01) and shorter hospital stay (7.6 days versus 12.1, *p* < 0.001) in MIS	No difference in terms of OS and PFS
Lee et al. [30]	2023	Korea	MIS vs. CT to assess PDS feasibility	Retrospective	614	Laparoscopic assessment PDS	Lower rate of suboptimal cytoreduction and lower postoperative morbidity in MIS group	No difference in terms of OS and PFS
Ceccaroni et al. [13]	2023	Italy	Feasibility of MIS in IDS	Retrospective, single-center	108	Laparoscopic IDS, PDS	R0: ~95%; major complications < 10%	3-yr OS: 66–84%; PFS not reported
Jorgensen et al. [31]	2023	USA	Compare MIS vs. laparotomy in IDS	Retrospective cohort	7897	Laparoscopic IDS	Shorter stay; lower 30- and 90-day mortality in MIS	OS: 35.9 vs. 37.4 mo (not significant)
Rauh-Hain et al. [32]	2023	International	Evaluate feasibility of RCT comparing MIS vs. open IDS after NAC	Randomized pilot trial	100	Laparoscopic IDS	R0: 88% (MIS) vs. 83% (open); conversion < 12.5%; better peri-op profile	OS/PFS not yet reported
Tozzi et al. (ULTRA-LAP) [12]	2023	Italy	Test safety, side effects and efficacy of laparoscopic Visceral-Peritoneal Debulking	Non-randomized prospective clinical trial	208	Laparoscopic IDS, PDS	Intra- and post-operative morbidity was very low in the MIS group. CR rate was 98% in MIS group and 94% in LPT group.	OS/PFS not yet reported
Uwins et al. (MIRRORS) [33]	2024	UK	Feasibility and safety of robotic IDS	Prospective cohort study	24	Robotic IDS	All patients achieved R < 1 (robotic R0 = 47.4%, open R0 = 0%). No patients had conversion to open	OS/PFS not yet reported

MIS: minimally invasive surgery; IDS: interval debulking surgery; PDS: primary debulking surgery; OS: overall survival; PFS: progression-free-survival, NACT: neoadjuvant chemotherapy; R0: no residual tumor.

## Data Availability

All data generated or analyzed during this study are included in this published article. Further information is available from the corresponding authors upon reasonable request.

## Abbreviations

The following abbreviations are used in this manuscript:

EOC	Epithelial ovarian cancer
PDS	Primary debulking surgery
IDS	Interval debulking surgery
MIS	Minimally invasive surgery
OS	Overall survival
PFS	Progression-free survival
NACT	Neoadjuvant chemotherapy
R0	no residual tumor
